# Determination of Aflatoxin B_1_ and B_2_ in Vegetable Oils Using Fe_3_O_4_/rGO Magnetic Solid Phase Extraction Coupled with High-Performance Liquid Chromatography Fluorescence with Post-Column Photochemical Derivatization

**DOI:** 10.3390/toxins11110621

**Published:** 2019-10-26

**Authors:** Li Yu, Fei Ma, Liangxiao Zhang, Peiwu Li

**Affiliations:** 1 Oil Crops Research Institute, Chinese Academy of Agricultural Sciences, Wuhan 430062, China; yuli01@caas.cn (L.Y.); mafei01@caas.cn (F.M.); zhanglx@caas.cn (L.Z.); 2 Key Laboratory of Biology and Genetic Improvement of Oil Crops, Ministry of Agriculture and Rural Affairs, Wuhan 430062, China; 3 Key Laboratory of Detection for Mycotoxins, Ministry of Agriculture and Rural Affairs, Wuhan 430062, China; 4 Laboratory of Quality and Safety Risk Assessment for Oilseeds Products (Wuhan), Ministry of Agriculture and Rural Affairs, Wuhan 430062, China; 5 Quality Inspection and Test Center for Oilseeds Products, Ministry of Agriculture and Rural Affairs, Wuhan 430062, China

**Keywords:** aflatoxin, magnetic solid phase extraction, graphene, high-performance liquid chromatography fluorescence, vegetable oil

## Abstract

In this study, magnetic graphene nanocomposite Fe_3_O_4_/rGO was synthesized by facile one-pot solvothermal method. The nanocomposite was successfully used as magnetic solid phase extraction (MSPE) adsorbents for the determination of aflatoxins in edible vegetable oils through the π–π stacking interactions. MSPE parameters including the amount of adsorbents, extraction and desorption time, washing conditions, and the type and volume of desorption solvent were optimized. Under optimal conditions, good linear relationships were achieved. Limits of detection of this method were as low as 0.02 µg/kg and 0.01 µg/kg for aflatoxin B_1_ and B_2_, respectively. Finally, the magnetic graphene nanocomposite was successfully applied to aflatoxin analysis in vegetable oils. The results indicated that the recoveries of the B-group aflatoxins ranged from 80.4% to 106.0%, whereas the relative standard deviations (RSDs) were less than 8.1%. Owing to the simplicity, rapidity and efficiency, Fe_3_O_4_/rGO magnetic solid phase extraction coupled with high-performance liquid chromatography fluorescence with post-column photochemical derivatization (Fe_3_O_4_/rGO MSPE-HPLC-PCD-FLD) is a promising analytical method for routine and accurate determination of aflatoxins in lipid matrices.

## 1. Introduction

Recently, edible vegetable oils have gained immense popularity over animal-based fats, which is attributed to their nutritional and health-promoting characteristic [1]. Vegetable oils meet dietary demands by providing energy and by transporting fat-soluble vitamins and antioxidant compounds which are widely used in home cooking and the food industry. However, the majority of the edible oilseeds, such as peanut, soybean and maize are easily attacked by *Aspergillus* strains, namely *A. flavus* and *A. parasiticus*. The secondary metabolites of those fungi are mainly aflatoxin B_1_ (AFB_1_) and B_2_ (AFB_2_), which can cause carcinogenic, teratogenic, mutagenic, immune-suppressive and estrogenic effects that are harmful to the human health [2,3]. The International Agency for Research on Cancer (IARC) of the world Health Organization (WHO) has classified AFB_1_ as carcinogenic to humans in 1993 [4].

To avoid hazardous symptoms to humans and animals, various countries have established specific regulations and prevention guidelines for aflatoxin management. The European Union has set strict standards for aflatoxins in groundnuts and other oilseed, in which the maximum levels (MLs) are 2 μg/kg for AFB_1_ and 4 μg/kg for the total aflatoxin concentration (AFTs). In Japan, the ML for AFB_1_ is 10 μg/kg in all foods, whereas the ML for peanuts and nuts is 10 μg/kg in Korea. In China, the MLs of AFB_1_ are set at 20 μg/kg for peanut and maize oils, and at 10 μg/kg for the other vegetable oils [5,6,7,8]. Owing to the current regulations and the survey requirements in edible oils, it is important to develop simple and sensitive methods for the detection of aflatoxins in complex matrices.

Various methods have been developed for the determination of AFTs in different matrices, including thin layer chromatography (TLC) [9], enzyme-linked immunosorbent assays (ELISA) [10] and high-performance liquid chromatography coupled with fluorescence (FLD)/mass spectrometry (MS) detection [11,12]. According to the ‘‘gold standard” for trace amount AFT detection, HPLC-FLD is the main method used for routine quantification of this type of compounds in a complex matrix. To maintain the natural fluorescence of AFTs, chemical and photochemical derivatization (PCD) has been used to avoid the emission quenching in the aqueous mobile phase. The PCD method dramatically increases the fluorescence signal of AFTs by the UV irradiation of the hydroxyl radical, which is in compliance with the guidelines of the green preparation chemistry including the improvement of the automatic manipulator, the absence of derivatizing reagents and the lower requirement for detection maintenance.

The extraction and enrichment procedure used for edible oils plays important roles in the accurate quantification of AFTs due to their low concentration in the triacylglycerol matrix. The extraction methods have been optimized including liquid–liquid extraction (“dilute-and-shoot” method) [13], solid phase extraction (SPE) [14], QuEChERS [15], gel permeation chromatography (GPC) [16], matrix solid phase dispersion (MSPD) [17] and cloud point extraction (CPE) [18]. However, the majority of these extraction methods used for aflatoxins in oil samples require tedious and time-consuming procedures and large volumes of organic solvents, and result in limited cycles of interface phase between the analytes and the extract absorbents. Therefore, it is necessary to develop a simple, rapid and accurate method for the determination of aflatoxins in vegetable oil.

Recently, magnetic solid-phase extraction (MSPE) has attracted particular interest with regard to sample pretreatment. The magnetic adsorbents are uniformly dispersed and agglomerated in extract solutions by external magnetic field. The MSPE procedure could evidently improve the interface phase between the adsorbents and the extractant by increasing the mass transfer coefficients of the analytes [19]. The structure of MSPE adsorbents mainly consists of magnetic carriers and functional groups, which play key roles in the enrichment process and affect the performance of the detection method. The functional activity of the MSPE composites could be prepared by chemical modification including metal [20], oxidative metal [21], silica [22] and metal-organic frameworks [23], or the composite synthesis containing magnetic nanoparticles combined with antibodies [24], molecularly imprinted polymers [25], carbon nanotubes [26] and graphene (G) [27].

Among the various materials applied as MSPE adsorbents, G and its derivative possess the single-layer/few-layer of *sp*^2^ hybridized carbon motif in the honeycomb lattice, which contributes to ultra-high surface area and a large delocalized π-electronic carbon network. Owing to this unique characteristic, G-based magnetic composites have been widely applied in the separation and purification of organic contaminants, biological macromolecules and heavy metals [28]. Reduced graphene oxide (rGO) possesses large amounts of polar groups with oxygen atoms, including hydroxyl, epoxy and carboxyl groups, which exhibit optimal adsorption capacity toward the oxygen and nitrogen functional groups of the organic pollutants by the interaction of dative bonds, cation-π interactions, electrostatic interactions or hydrogen bonds compared with the corresponding adsorption capacity noted in non-polar and hydrophobic graphene adsorbents. Previous studies indicated that graphene oxide (GO) materials could be used as simple, rapid and cost-effective dSPE adsorbents to extract aflatoxins from peanut samples [29]. However, rGO materials are easily aggregated in extract solutions and difficult to retrieve from the suspension owing to ultra-light and hydrophilic properties. To prevent rGO aggregation and facilitate rGO-dSPE, the chemical fabrication of rGO and Fe_3_O_4_ can be used to synthesize an rGO hybrid magnetite. This is a promising technological method used in the enrichment of AFTs from complex matrices with magnetic separation.

In the present study, magnetic graphene Fe_3_O_4_/rGO adsorbents were characterized and applied to extract AFB_1_ and AFB_2_ from vegetable oils. The adsorbents were coupled with high performance liquid chromatography fluorescence detection (HPLC-FLD) analysis. Fe_3_O_4_/rGO adsorbents were synthesized with high yield via the facile one-pot solvothermal method. The magnetic graphene-based adsorbents indicated optimal adsorption capacity toward AFTs due to the presence of π–π interactions and hydrogen bonds. Furthermore, the adsorbents were washed with non-polar hexane to completely remove triglyceride matrix components. Following post-column derivatization (PCD) coupled with FLD detection, a rapid, simple and accurate Fe_3_O_4_/rGO MSPE-HPLC-PCD-FLD method was developed for the determination of AFB_1_ and AFB_2_ in vegetable oil samples.

## 2. Results and Discussion

### 2.1. Characterization of Fe_3_O_4_/rGO Adsorbents

**Figure 1:**
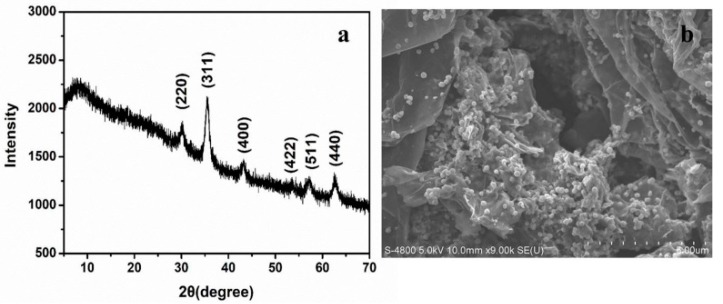
XRD spectrum (**a**) and SEM image (**b**) of Fe_3_O_4_/rGO.

**Figure 2:**
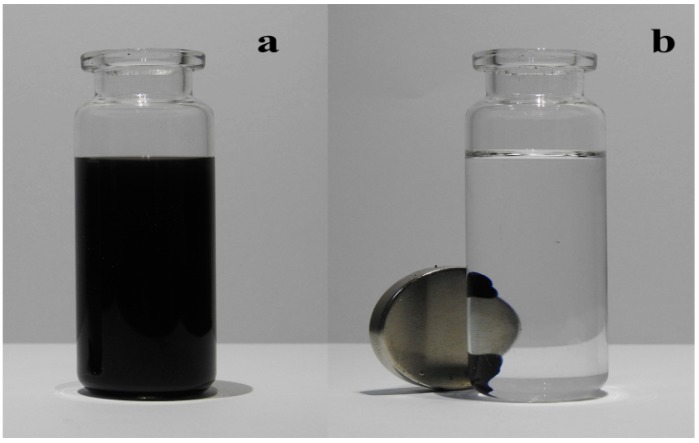
The dispersed and magnetic characteristic of the Fe_3_O_4_/rGO adsorbents in matrix solutions (**a**) and collected by external magnet field (**b**).

The synthesized adsorbent material was characterized by X-ray diffraction (XRD) and scanning electron microscopy (SEM). In Figure 1a, the characteristic peak of GO that was located at 2θ around 11° could not be observed, which was attributed to the reduction of GO. No distinct peak was observed in the range of 2θ from 5° to 30°, indicating that the resulting rGO was very poorly ordered along the stacking direction. The significant diffraction peaks of the sample ranged from 30° to 70° (2θ) and could be assigned to the crystal Fe_3_O_4_ cubic structure (JCPDS 19-0629). As shown in Figure 1b, the surface of the rGO nanosheets was decorated with monodisperse nanoparticles, and the size of the Fe_3_O_4_ nanoparticles was approximately 200 nm. Furthermore, rGO nanosheets containing several wrinkles and folds were observed, indicating that the stacking of rGO nanosheets was disordered due to the insertion of the Fe_3_O_4_ nanoparticles. Figure 2 showed that the synthesized adsorbent material with good dispersibility can be easily collected by an external magnet field in the organic solvent.

### 2.2. Optimization of MSPE Conditions

In order to improve the extraction efficiency, several parameters were investigated including the adsorbent amount, the extraction time, the volume of washing solvent, the desorption time, the desorption solvent and its volume. The optimization of the MSPE conditions was performed using 2.0 g non-contaminated oil samples spiked with 5 μg/kg for AFB_1_ and AFB_2_, respectively. The recoveries were calculated by the ratios of the chromatographic peak of the analytes to those of the AFT standards.

#### 2.2.1. Amount of Fe_3_O_4_/rGO Adsorbents

**Figure 3:**
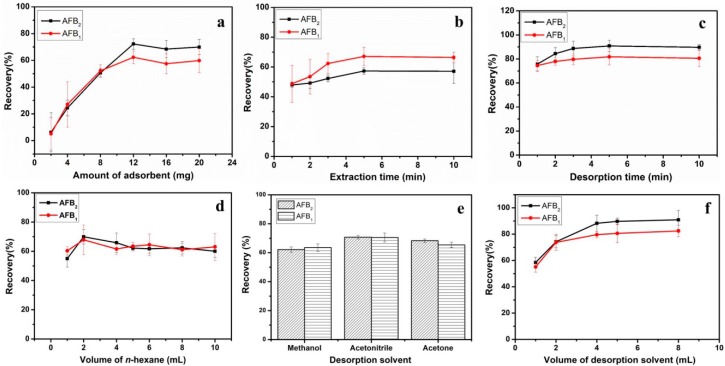
Effects of the key parameters on the recovery of aflatoxins (*n* = 3), including the amount of Fe_3_O_4_/rGO (**a**), extraction time (**b**), desorption time (**c**), volume of *n*-hexane (**d**), desorption solvent (**e**), and volume of elution solvent (**f**).

To achieve optimal recovery efficiency toward aflatoxins, different amounts of Fe_3_O_4_/rGO adsorbents (2, 4, 8, 12, 16 and 20 mg) were added to the extract and the analytes were purified from the lipid samples. As shown in Figure 3a, the recoveries of AFB_1_ and AFB_2_ increased dramatically following an increase in the amount of adsorbent from 2 to 12 mg, and subsequently remained constant regardless of the further increase in the concentration of the Fe_3_O_4_/rGO MSPE adsorbents. Ultimately, 12 mg was applied as the amount of the adsorbent for the following experiments.

#### 2.2.2. Extraction and Desorption Time

The extraction and desorption time ranged from 1 to 10 min, respectively. In Figure 3b, the increase in the extraction time from 1 to 5 min caused a gradual increase in the extraction efficiency. Moreover, the dynamic process for the adsorption of AFTs was equilibrated synchronously. Therefore, the extraction time was set at 5 min for the rapid and efficient purification of AFTs owing to the high interfacial surface between the adsorbents and the diluted oil sample, and due to the increase in the mass transfer coefficients during the extraction procedure. In Figure 3c, the same variation tendency of desorption efficiency was noted, and the time period of 3 min was set to for the elution of the retained AFTs from the Fe_3_O_4_/rGO adsorbents.

#### 2.2.3. Washing Conditions

When the analytes were loaded on MSPE adsorbents, the matrix interferences were eliminated by washing step notably for the complex lipophilic samples [30]. The polarity of the washing solvent requires optimal miscibility with the matrix component triglycerides in order to avoid the loss of AFTs. The double bond of the terminal furan ring, the phenyl, and the carbonyl moiety are the hydrophobic and hydrophilic structures of AFTs, which could be easily adsorbed by Fe_3_O_4_/rGO adsorbents via the π–π interactions and the formation of hydrogen bonds with the MSPE adsorbents. The non-polar solvent *n*-hexane was selected and applied to the washing step to remove the triglycerides from the matrix, due to its low polarity and its ability to disrupt hydrophilic interactions. The influence of *n*-hexane volume on the recovery of AFTs was optimized in the range of 1 to 10 mL. As shown in Figure 3d, no significant changes in the recoveries were found following 2 mL of *n*-hexane use for purification. Therefore, 2 mL of *n*-hexane was selected in the washing procedure.

#### 2.2.4. Desorption Conditions

It is vital to increase the efficiency of desorption conditions in order to disrupt the interactions between the extracted AFTs and the surface of the Fe_3_O_4_/rGO adsorbents. Therefore, it is essential to optimize the type and volume of the desorption solvent for optimal analytical performance. The recoveries of methanol, acetonitrile and acetone as desorption solvents were present in Figure 3e. The high polarity of the acetonitrile solvent was more efficient in disrupting the π–π interactions and the hydrogen bonds with the MSPE adsorbents compared with that noted in the methanol and acetone solvents. In addition, the effects of the desorption volume range (1–8 mL) were evaluated. In Figure 3f, the recoveries of AFTs increased dramatically when the volume was increased from 1 to 4 mL, and no significant changes were noted afterwards. Therefore, 4 mL of acetonitrile was selected in the desorption process.

### 2.3. Method Validation

#### 2.3.1. Matrix Effect

The matrix of the lipid samples could enhance or diminish the FLD intensity of the analytes and affect accuracy and reproducibility of the analysis method. The post-extraction spiked method was used to assess the matrix effects by comparing the calibration slopes between the lipid matrix and the pure standard solvent. The calibration curves were constructed by plotting the area against the concentration of the analytes. The matrix effect of Fe_3_O_4_/rGO MSPE-HPLC-PCD-FLD was evaluated by the following Equation (1):

(1) $$\mathrm{Matrix}\;\mathrm{Effect}\;\left(\%\right)=\frac{\mathrm{Slope}\;\mathrm{in}\;\mathrm{solvent}-\mathrm{Slope}\;\mathrm{in}\;\mathrm{matrix}}{\mathrm{Slope}\;\mathrm{in}\;\mathrm{solvent}}\times 100\%$$

The matrix effect of AFB_1_ and AFB_2_ were 14.9% and 12.5%, respectively. The results indicated that the MSPE purification step could not remove the lipid matrix completely. Therefore, the matrix-matched calibration curve was applied for the accurate quantification of the AFTs in oil samples.

#### 2.3.2. Linearity, Accuracy and Precision of the Method

Linearity was evaluated through the matrix-matched calibration at six different concentration and the correlation coefficient (*R^2^*) was constructed by the linear regression equation. As listed in Table 1, *R*^2^ were higher than 0.9967 for AFTs; besides, the standard deviation of the residuals was less than 20%, indicating satisfactory linearity. The limit of detection (LOD) and the limit of quantification (LOQ) for AFTs were evaluated by the signal-to-noise ratio of the FLD chromatogram for oil samples (S/N = 3 for LOD and S/N = 10 for LOQ), respectively. The results showed that the LOD and LOQ were 0.02 μg/kg and 0.10 μg/kg for AFB_1_, and 0.01 μg/kg and 0.10 μg/kg for AFB_2_ respectively, which could meet the strict regulatory levels set in vegetable oil by the National Criterion of China (10 µg/kg for AFB_1_).

The reproducibility was evaluated by the intra-day and inter-day precision as relative standard deviation (RSD), which were validated by AFTs spiked at five different concentration (0.1, 0.5, 1.0, 2.0, 20 µg/kg) in blank peanut oil. Six parallel extractions of oil sample within one day were obtained the intra-day RSDs, and the inter-day RSDs were tested by extracting AFTs from spiked oil samples that were prepared independently in four individual days. The results showed that the intra- and inter-day RSDs were less than 8.7% and 10.5%, respectively. Therefore, the developed method had the acceptable repeatability for routine analysis.

**Figure 4:**
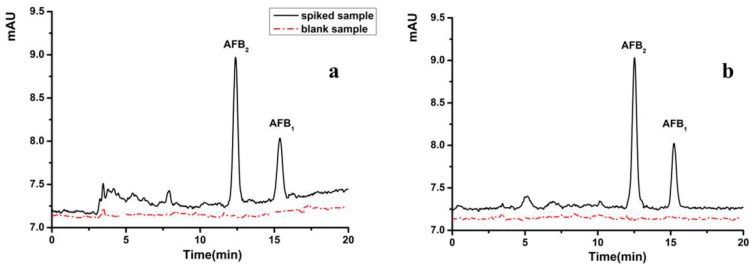
Typical chromatograms of aflatoxin analysis in peanut (**a**) and almond (**b**) oils. (AFB_1_ and AFB_2_ spiked at 1.0 µg/kg and 0.5 µg/kg, respectively.).

The accuracy and reliability of the methods were evaluated by spiking AFB_1_ and AFB_2_ in the blank oil samples. The recovery was measured by comparing the concentration of the analytes calculated from the matrix-matched calibration curve with the spiked concentration accordingly. The typical chromatographs of the HPLC-PCD-FLD for AFB_1_ and AFB_2_ are shown in Figure 4, and the recovery of analyte detection in the variety of the vegetable oils is summarized in Table 2. The recovery of the analytes were in the range of 80.4–106.0% and the RSDs were less than 8.1%, illustrating optimal accuracy and reliability of the method.

#### 2.3.3. Analysis of Real Samples

The Fe_3_O_4_/rGO MSPE-HPLC-PCD-FLD method was applied to analyze the concentration of AFB_1_ and AFB_2_ in 82 vegetable oils from the supermarkets in Wuhan (China), including 15 corn oils, 15 peanut oils, 12 soybean oils, 12 rapeseed oils, 12 rice oils, 8 walnut oils and 8 almond oils. The results indicated the absence of positive samples. A trace amount of AFB_1_ 0.7 μg/kg was found in only one peanut oil sample.

A comparative study of this proposed method was performed for the determination of AFB_1_ and AFB_2_ in vegetable oils and the results were shown in Table 3. The proposed method possessed optimal accuracy and recovery and excellent reproducibility. The complete Fe_3_O_4_/rGO MSPE steps could be achieved in 15 min and were directly analyzed, which could avoid laborious purification steps and time-consuming chemical derivatization. The sensitivity and selectivity of the proposed PCD-HPLC-FLD method were comparable with the sophisticated methodology using LC-MS/MS for aflatoxins. In addition, this was the first study that examined the application of the Fe_3_O_4_/rGO MSPE adsorbents for the extraction of AFB_1_ and AFB_2_ from vegetable oils. The method demonstrated high potential for simple, rapid and environmentally friendly pretreatment in complex fatty matrix.

## 3. Conclusions

In the present study, Fe_3_O_4_/rGO MSPE adsorbents were synthesized and used for the extraction and purification of AFB_1_ and AFB_2_ from vegetable oils. The characterization data indicated that rGO nanosheets were coated with Fe_3_O_4_ nanoparticles and possessed uniform size and shape. Owing to their unique features, the Fe_3_O_4_/rGO MSPE adsorbents were used for the enrichment and eliminating the presence of interfering substances in oils. Limits of detection of this method were as low as 0.02 µg/kg and 0.01 µg/kg for AFB_1_ and AFB_2_, respectively. The recovery of the analytes was in the range of 80.4%–106.0% and the RSDs less than 8.1%, which suggested optimal accuracy and reliability for the routine determination of aflatoxins in a variety of vegetable oils. Therefore, the Fe_3_O_4_/rGO MSPE-HPLC-PCD-FLD could be applied as a promising analytical method for simple, rapid and accurate quantification of organic contaminants in complex matrices.

## 4. Materials and Methods

### 4.1. Chemicals and Materials

Graphite flakes (~150 μm flakes), AFB_1_ and AFB_2_ standards were purchased from Sigma-Aldrich Co. (St. Louis, MO, USA). HPLC-grade methanol (MeOH), acetonitrile and acetone were supplied by Fisher Chemical Co. (Geel, Antwerp, Belgium). Potassium permanganate (KMnO_4_), hydrochloric acid (HCl, 37%), sulfuric acid (H_2_SO_4_, 98%), hydrogen peroxide (H_2_O_2_, 30%), phosphoric acid (H_3_PO_4_, 85%), ethylene glycol (EG), ethanol, ferric chloride (FeCl_3_), sodium acetate trihydrate (NaAc) and *n*-hexane were of analytical grade and were purchased from Sinopharm Chemical Reagent Co. (Shanghai, China). Unless otherwise stated, all other inorganic chemicals and organic solvents were of analytical reagent grade or higher. The water used was purified with the Milli-Q system from Millipore Co. (Billerica, MA, USA).

A mixed stock solution was prepared with methanol and stored at −20 °C in the dark. A series of standard solutions were prepared by diluting the stock solution with methanol to appropriate concentrations. All the standard solutions were stored at 4 °C in the dark. The stability of AFB_1_ and AFB_2_ (0.10 µg/kg) were all evaluated as those standard solution kept in the auto-sampler at 4 °C (48 h) and the CV of six injections were 3.7% and 4.1%, respectively.

### 4.2. Apparatus

An ultrasonic instrument KQ-800KDE (Kunshan Ultrasound Instrument Co., Kunshan, China) and a high-speed centrifuge CF16RXII (Hitachi Co., Tokyo, Japan) were used for GO preparation. Chromatographic analyses were performed on an Agilent 1100 HPLC-FLD system equipped with a photochemical post-column derivatization reactor (Pribolab Pte. Ltd., Singapore). The X-ray diffraction (XRD) experiment was performed on a X’Pert powder diffractometer (PANalytical Co., Almelo, The Netherlands) with a Cu Kα radiation (λ = 1.5418 Å) and a graphite monochromator. The diffraction data were recorded for 2θ between 0.5° and 70° with a resolution of 0.033°. The size and morphology of the magnetic nanoparticles were observed by a Hitachi S-4800 scanning electron microscope (Hitachi Ltd., Tokyo, Japan).

### 4.3. Synthesis of Fe_3_O_4_/rGO Adsorbents

GO was prepared from graphite flakes using KMnO_4_ and a 9:1 mixture of concentrated H_2_SO_4_/H_3_PO_4_ as oxidizing agents by a method reported in our previous study [29]. The Fe_3_O_4_/rGO nanocomposite was synthesized via a facile one-pot solvothermal method [35]. In a typical process, 400 mg of GO was mixed in 60 mL of EG and was homogenized for 4 h under ultrasonic vibrations in order to produce a homogeneous solution. Subsequently, 0.65 g of FeCl_3_ was dissolved in the GO containing solution. Homogenization was achieved by ultrasonic vibrations for 20 min. 2.6 g of NaAc was added into the aforementioned solution, which was vigorously stirred for 30 min to ensure that the precursor was dissolved in the solution completely. Finally, the mixed solution was transferred into a teflon-lined stainless-steel autoclave, maintaining a set temperature of 200 °C for 8 h. When the autoclave was cooled down to room temperature, the obtained black product was filtered, washed with ethanol for several times and dried in vacuum.

### 4.4. Sample Preparation

Different types of edible vegetable oils, including rapeseed oil, peanut oil, corn oil, soybean oil, walnut oil, rice oil and almond oil, were purchased from local markets (Wuhan, China). All the oil samples were stored at room temperature.

### 4.5. Magnetic Solid-Phase Extraction Procedure

**Figure 5:**
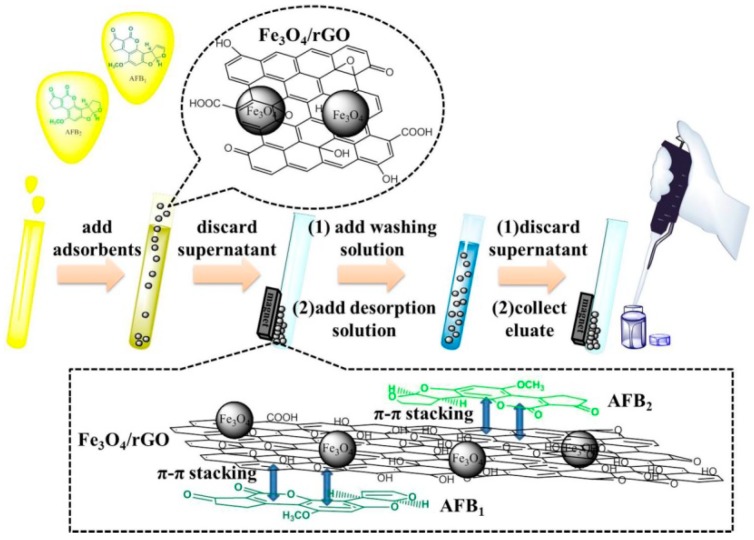
The schematic illustration of MSPE based on the magnetic graphene nanocomposite.

The MSPE procedure of AFB_1_ and AFB_2_ from the oil samples was illustrated in Figure 5. Initially, 2.0 g (± 0.001 g) of oil sample was weighed accurately and diluted with 10 mL of *n*-hexane. Subsequently, 12.0 mg of Fe_3_O_4_/rGO adsorbents were added to the mixture and vigorously vortexed for 5 min. A powerful magnet was applied to the bottom of the tube to attract and isolate the magnetic graphene adsorbent, and the supernatant was discarded. A total of 2 mL of *n*-hexane was used for washing in order to remove the interfering compounds in the lipid matrix by vortexing for 60 s. Finally, 4 mL of acetonitrile was used for the desorption and was added by ultrasonic agitation for 3 min. The desorption solution was evaporated under a mild stream of N_2_ at 40 °C and reconstitued with 100 μL H_2_O/MeOH (55:45, *v*/*v*).

### 4.6. HPLC-PCD-FLD Analytical Conditions

The chromatographic separation was performed on a Kromasil C_18_ column (150 mm × 4.6 mm, 5 μm particle size) using a H_2_O/MeOH (55:45, *v*/*v*) mobile phase at a flow rate of 0.8 mL/min with a total running time of 20 min. The detection wavelengths were set at 360 nm and 440 nm for the excitation and emission, respectively. The column temperature was set at 30 °C and the injection volume was adjusted to 10 μL.

### 4.7. Statistical Analysis

All the vegetable oils were analyzed in triplicate, and the results were reported as average ± standard deviation (SD). The statistical analyses were performed using the @Risk 5.5.1 software package from Palisade Co. (Australia, 2010). Significant differences were determined by the Student *t*-test at a significance level of 0.05 (*p* < 0.05).

## Figures and Tables

**Table 1:** Linear range and equation, correlation coefficient (*R^2^*), limits of detection (LOD), limits of quantification (LOQ) and precision for the determination of aflatoxins by Fe_3_O_4_/rGO magnetic solid phase extraction coupled with high-performance liquid chromatography fluorescence with post-column photochemical derivatization (Fe_3_O_4_/rGO MSPE-HPLC-PCD-FLD).

Analyte	Liner Range (μg/kg)	Linear Equation	*R^2^*	LOD (µg/kg)	LOQ (µg/kg)	Intra-Day Precision (RSD %, *n* = 6)					Inter-Day Precision (RSD %, *n* = 4)				
						0.1	0.5	1.0	2.0	20	0.1	0.5	1.0	2.0	20
AFB_1_	0.10–25	*y* = 130.686*x* (±11.029) − 2.31921 (±1.14467)	0.9967	0.02	0.10	8.7	4.5	4.8	3.7	2.3	10.5	9.8	6.2	3.9	3.2
AFB_2_	0.10–20	*y* = 480.364*x* (±10.605) + 1.55194 (±0.43423)	0.9978	0.01	0.10	7.3	7.5	4.9	2.3	1.3	9.8	9.0	5.1	5.8	3.9

**Table 2:** Recovery and precision of AFB_1_ and AFB_2_ in vegetable oil samples ^a.^

Analyte	Recovery (%, *n* = 3) ^b^							
	Corn Oil	Soybean Oil	Rapeseed Oil	Rice Oil	Almond Oil	Peanut Oil I	Peanut Oil II	Peanut Oil III
AFB_1_	86.3 (5.3)	88.7 (5.0)	80.4 (4.6)	82.1 (5.6)	96.6 (7.7)	88.7 (6.7)	94.1 (6.4)	93.2 (8.1)
AFB_2_	105.8 (6.3)	102.6 (6.7)	98.1 (2.8)	100.6 (3.2)	103.5 (4.2)	106.0 (4.4)	103.2 (3.5)	95.3 (6.1)

^a^ The concentration of AFB_1_ and AFB_2_ were spiked at 1 μg/kg and 0.5 μg/kg, respectively. ^b^ The analyzed data were the mean ± standard deviation.

**Table 3:** Comparison of pretreatment procedures, LOQ and recovery for the determination of AFB_1_ and AFB_2_ in edible oils. Magnetic solid-phase extraction, MSPE.

Adsorbents	Pretreatment	Pretreatment Time (min)	Derivatization Condition	Determination	Recovery (%)	LOQs (μg/kg)	Reference
-	Dispersive liquid-liquid micro-extraction after IAC clean up	<120	-	LC-FLD	96–109.9	2.8 × 10^3^ (AFB_1_) 0.4 × 10^3^ (AFB_2_)	[31]
	IAC clean up	<30	-	UPLC-MS/MS	90–105	0.12–0.15 (AFB_1_)	[32]
-	Supercritical fluid chromatography	15	-	UPC^2^-MS/MS	98, 104	0.05 (AFB_1_) 0.08 (AFB_2_)	[33]
Humic acid-bonded silica	SPE	8–10	-	HPLC-MS/MS	82–106	0.044 (AFB_1_) 0.057 (AFB_2_)	[14]
C_18_, PSA & neutral Al_2_O_3_	QuEChERS	38	-	HPLC-MS/MS	83–100.3	0.18 (AFB_1_) 0.13 (AFB_2_)	[15]
-	Dispersive liquid-liquid micro-extraction	<20	In situ chemical derivatization	HPLC-FLD	91.8–121.5	0.10 (AFB_1_) 0.017 (AFB_2_)	[34]
rGO-Fe_3_O_4_	MSPE	15	photochemical derivatization	HPLC-FLD	80.38–109.03	0.10 (AFB_1_) 0.10 (AFB_2_)	This work

## References

[1] Yang R., Zhang L., Li P., Yu L., Mao J., Wang X., Zhang Q. (2018). A review of chemical composition and nutritional properties of minor vegetable oils in China. Trends Food Sci. Technol..

[2] Bhat R., Reddy K.R.N. (2017). Challenges and issues concerning mycotoxins contamination in oil seeds and their edible oils: Updates from last decade. Food Chem..

[3] Li P., Zhang Q., Zhang W. (2009). Immunoassays for aflatoxins. TrAC-Trend Anal. Chem..

[4] World Health Organization, International Agency for Research on Cancer (1993). Aflatoxins. IARC Monographs on the Evaluation of Carcinogenic Risks to Humans.

[5] European Commission (2006). (EC) Commission Regulation (EC) 1881/2006 of 19 December 2006 setting maximum levels for certain contaminants in foodstuffs. Off. J. Eur. Union.

[6] European Commission (2010). (EC) Commission Regulation (EC) 165/2010 of 26 February 2010 amending Regulation (EC) No. 1881/2006 setting maximum levels for certain contaminants in foodstuffs. Off. J. Eur. Union.

[7] Wu L.X., Ding X.X., Li P.W., Du X.H., Zhou H.Y., Bai Y.Z., Zhang L.X. (2016). Aflatoxin contamination of peanuts at harvest in China from 2010 to 2013 and its relationship with climatic conditions. Food Control.

[8] National Criterion of China (2017). Maximum Levels of Mycotoxins in Foods.

[9] Scott P.M., Lawrence J.W., Van Walbeek W. (1970). Detection of mycotoxins by thin-layer chromatography: Application to screening of fungal extracts. Appl. Microbiol..

[10] Lee N.A., Wang S., Allan R.D., Kennedy I.R. (2004). A rapid aflatoxin B_1_ ELISA:  Development and validation with reduced matrix effects for peanuts, corn, pistachio, and soybeans. J. Agric. Food Chem..

[11] Cavaliere C., Foglia P., Guarino C., Nazzari M., Samperi R., Laganà A. (2007). Determination of aflatoxins in olive oil by liquid chromatography-tandem mass spectrometry. Anal. Chim. Acta.

[12] Huertas-Pérez J.F., Arroyo-Manzanares N., Hitzler D., Castro-Guerrero F.G., Gámiz-Gracia L., García-Campaña A.M. (2018). Simple determination of aflatoxins in rice by ultra-high performance liquid chromatography coupled to chemical post-column derivatization and fluorescence detection. Food Chem..

[13] Sheijooni-Fumani N., Hassan J., Yousefi S.R. (2011). Determination of aflatoxin B_1_ in cereals by homogeneous liquid-liquid extraction coupled to high performance liquid chromatography-fluorescence detection. J. Sep. Sci..

[14] Zhou N.Z., Liu P., Su X.C., Liao Y.H., Lei N.S., Liang Y.H., Zhou S.H., Lin W.S., Chen J., Feng Y.Q. (2017). Low-cost humic acid-bonded silica as an effective solid-phase extraction sorbent for convenient determination of aflatoxins in edible oils. Anal. Chim. Acta.

[15] Zhao H., Chen X., Shen C., Qu B. (2017). Determination of 16 mycotoxins in vegetable oils using a QuEChERS method combined with high-performance liquid chromatography-tandem mass spectrometry. Food Addit. Contam. Part A.

[16] Mahoney N., Molyneux R.J. (2010). Rapid analytical method for the determination of aflatoxins in plant-derived dietary supplement and cosmetic oils. J. Agric. Food Chem..

[17] Blesa J., Soriano J.M., Moltó J.C., Marín R., Mañes J. (2003). Determination of aflatoxins in peanuts by matrix solid-phase dispersion and liquid chromatography. J. Chromatogr. A.

[18] Lv J., Yang Y. (2013). Determination of aflatoxin B_1_ and B_2_ in peanut and peanut oil using cloud point extraction followed by ultra-high-performance liquid chromatography. J. Liq. Chromatogr. Relat. Technol..

[19] Ríos Á., Zougagh M. (2016). Recent advances in magnetic nanomaterials for improving analytical processes. TrAC-Trend Anal. Chem..

[20] Wang D., Duan H., Lü J., Lü C. (2017). Fabrication of thermo-responsive polymer functionalized reduced graphene oxide@Fe_3_O_4_@Au magnetic nanocomposites for enhanced catalytic applications. J. Mater. Chem. A.

[21] Peng H.P., Liang R.P., Qiu J.D. (2011). Facile synthesis of Fe_3_O_4_@Al_2_O_3_ core-shell nanoparticles and their application to the highly specific capture of heme proteins for direct electrochemistry. Biosens. Bioelectron..

[22] Zheng L., Pi F., Wang Y., Xu H., Zhang Y., Sun X. (2016). Photocatalytic degradation of acephate, omethoate, and methyl parathion by Fe_3_O_4_@SiO_2_@mTiO_2_ nanomicrospheres. J. Hazard. Mater..

[23] Lian L., Zhang X., Hao J., Lv J., Wang X., Zhu B., Lou D. (2018). Magnetic solid-phase extraction of fluoroquinolones from water samples using titanium-based metal-organic framework functionalized magnetic microspheres. J. Chromatogr. A.

[24] Condina M.R., Guthridge M.A., McColl S.R., Hoffmann P. (2009). A sensitive magnetic bead method for the detection and identification of tyrosine phosphorylation in proteins by MALDI-TOF/TOF MS. Proteomics.

[25] Ma R.T., Ha W., Chen J., Shi Y.P. (2016). Highly dispersed magnetic molecularly imprinted nanoparticles with well-defined thin film for the selective extraction of glycoprotein. J. Mater. Chem. B.

[26] Korneva G., Ye H., Gogotsi Y., Halverson D., Friedman G., Bradley J.C., Kornev K.G. (2005). Carbon nanotubes loaded with magnetic particles. Nano Lett..

[27] Thu T.V., Sandhu A. (2014). Chemical synthesis of Fe_3_O_4_-graphene oxide nanohybrids as building blocks for magnetic and conductive membranes. Mater. Sci. Eng. B-Adv. Funct. Solid-State Mater..

[28] Li N., Jiang H.L., Wang X., Wang X., Xu G., Zhang B., Wang L., Zhao R.S., Lin J.M. (2018). Recent advances in graphene-based magnetic composites for magnetic solid-phase extraction. TrAC-Trend Anal. Chem..

[29] Yu L., Li P., Zhang Q., Zhang W., Ding X., Wang X. (2013). Graphene oxide: an adsorbent for the extraction and quantification of aflatoxins in peanuts by high-performance liquid chromatography. J. Chromatogr. A.

[30] Ma F., Li P., Zhang Q., Yu L., Zhang L. (2015). Rapid determination of *trans*-resveratrol in vegetable oils using magnetic hydrophilic multi-walled carbon nanotubes as adsorbents followed by liquid chromatography-tandem mass spectrometry. Food Chem..

[31] Afzali D., Ghanbarian M., Mostafavi A., Shamspur T., Ghaseminezhad S. (2012). A novel method for high preconcentration of ultra trace amounts of B_1_, B_2_, G_1_ and G_2_ aflatoxins in edible oils by dispersive liquid–liquid microextraction after immunoaffinity column clean-up. J. Chromatogr. A.

[32] Xie J., Peng T., He J.L., Shao Y., Fan C.L., Chen Y., Jiang W.X., Chen M., Wang Q., Pei X.Y. (2015). Preparation and characterization of an immunoaffinity column for the selective extraction of aflatoxin B_1_ in 13 kinds of foodstuffs. J. Chromatogr. B.

[33] Lei F., Li C., Zhou S., Wang D., Zhao Y., Wu Y. (2016). Hyphenation of supercritical fluid chromatography with tandem mass spectrometry for fast determination of four aflatoxins in edible oil. Rapid Commun. Mass Spectrom..

[34] Wang N., Duan C., Geng X., Li S., Ding K., Guan Y. (2019). One step rapid dispersive liquid-liquid micro-extraction with in-situ derivatization for determination of aflatoxins in vegetable oils based on high performance liquid chromatography fluorescence detection. Food Chem..

[35] Wu Q., Feng C., Wang C., Wang Z. (2013). A facile one-pot solvothermal method to produce superparamagnetic graphene-Fe_3_O_4_ nanocomposite and its application in the removal of dye from aqueous solution. Colloid Surf. B-Biointerfaces.

